# Supplementary material for: Role of the Fungus Pneumocystis in IL1β Pathway Activation and Airways Collagen Deposition in Elastase-Induced COPD Animals
Source: Int J Mol Sci. 2024 Mar 9;25(6):3150. doi: 10.3390/ijms25063150 (PMC10969872; doi:10.3390/ijms25063150)
Supplement: Supplementary file 1 [file ijms-25-03150-s001.zip › ijms-2886088-supplementary.pdf]

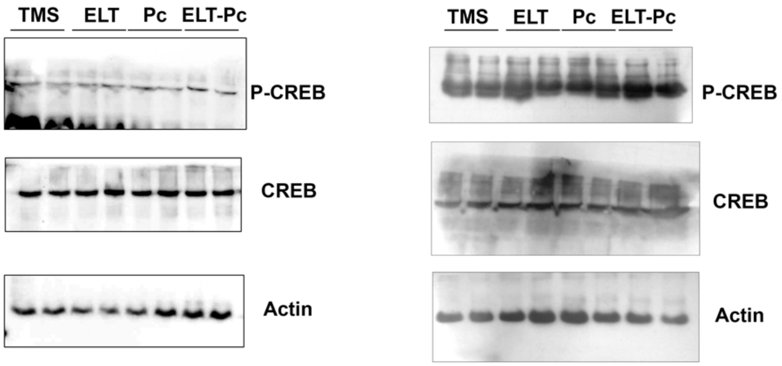

Figure S1: Detection of P-CREB. Protein levels of transcription factor CREB and the phosphorylated version. Two replicates of the experiment are indicated in this figure. Actin was used as an internal control. Quantification of these experiments is presented in Figure 3 of the main text of the manuscript.
